# Surgical Outcomes of 27-Gauge Pars PLana Vitrectomy for Symptomatic Vitreous Floaters

**DOI:** 10.1155/2017/5496298

**Published:** 2017-11-29

**Authors:** Zhong Lin, Rui Zhang, Qi Hua Liang, Ke Lin, Yu Shu Xiao, Nived Moonasar, Rong Han Wu

**Affiliations:** ^1^The Eye Hospital, School of Ophthalmology and Optometry, Wenzhou Medical University, Wenzhou, Zhejiang, China; ^2^Liaocheng People's Hospital of Shandong Province, Liaocheng, China; ^3^Ophthalmology Department, University of the West Indies, St. Augustine, Trinidad and Tobago

## Abstract

**Purpose:**

To report the surgical outcomes of 27-gauge pars plana vitrectomy (PPV) for symptomatic vitreous floaters.

**Methods:**

47 eyes of 47 patients (39 males, 83.0%) with symptomatic vitreous floaters who underwent 27-gauge PPV and followed up for more than 6 months were included. The mean age was 34.7 ± 13.5 years.

**Results:**

No operative complication occurred. At first day postoperatively, the intraocular pressure (IOP) was significantly lower than that at other time points (8.6 ± 2.7 mmHg, *p* < 0.001). 28 (59.6%) eyes had transient hypotony (IOP < 8 mmHg). All were recovered within 1 week postoperatively. The BCVA of 41 eyes (41/47, 87.2%) remained unchanged or improved. Postoperative complications occurred in two eyes: one (2.1%) had endophthalmitis and one (2.1%) had retinal detachment. No clinical significant cataract was observed in the 42 postoperative phakic eyes. 91.5% of the patients were satisfied with the surgery outcome. Besides, 91.3% of the patients felt that the floaters were removed completely or only had an acceptable residual.

**Conclusion:**

Visual acuity of most patients remained unchanged or improved following 27-gague pars plana vitrectomy for symptomatic vitreous floaters, resulting in high patient satisfaction. However, this treatment should be performed with great caution since severe postoperative complications may still occur. This trial is registered with NCT03049163.

## 1. Introduction

The human vitreous body consists of approximately 98% water and macromolecules including collagen and hyaluronic acid [1]. Vitreous opacities occur most commonly as a result of age-related and/or myopia-induced alteration in the vitreous composition and organization, leading to liquefaction (synchysis) and collapse (syneresis) of the vitreous body [2]. This will be observed by patients as hair-like, fly-like gray linear obscurations to vision that move with ocular saccades and demonstrate an overdamping behavior described by patients as “floating” in and out of their vision.

Though not have been considered as a severe medical problem by most ophthalmic practitioners, recent studies found that floaters are perceived by patients as a serious medical condition that has a significant negative impact on their vision and quality of life [3–5]. Though neodymium-doped yttrium aluminum garnet (Nd:YAG) laser vitreolysis is also considered [6, 7], small gauge vitrectomy seems to be the only effective therapy of symptomatic floaters at present [8–12].

The 27-gauge pars plana vitrectomy (PPV) was reported to be safe and effective for various vitreoretinal diseases [13, 14], including vitreous opacity [14]. The use of smaller gauge of vitrectomy instruments was reported to reduce the risk of retinal detachment (RD) from 4.9% (20G instruments) to 1.1% (23G) [15]. Recently, Sebag et al. reported that using 25-gauge vitrectomy instruments and not inducing posterior vitreous detachment can be uniformly safe for systemic vitreous floaters, since apparently lower incidence (none) of retinal tear or infection was observed. Hence, there is clinical significance to know the surgical outcomes of 27-gauge vitrectomy for symptomatic vitreous floaters.

## 2. Methods

47 eyes of 47 patients (39 males, 83.0%), who underwent 27-gauge PPV for symptomatic vitreous floaters from June 2015 to July 2016 in the Eye Hospital of Wenzhou Medical University and followed up for at least 6 months, were included in this study. The inclusion criteria of this study were as follows: (1) age > 18 years; (2) subjective sensation of the “floaters” which disturbed his/her life moderately or severely for more than 3 months; (3) clinic examination showed the vitreous opacity crumb. The exclusion criteria were as follows: (1) patients who had vitrectomy surgery before; (2) patients who had penetrating ocular trauma before. The study followed the tenets of the Declaration of Helsinki and was approved by the Ethics Committee of the Eye Hospital of Wenzhou Medical University. All participants signed a written and informed consent.

Table 1 presented the detail baseline characters of these patients. The mean age was 34.7 ± 13.5 (range 18–64) years. 35 patients (74.5%, 35/47) were younger than 40 years. The median time of the subjective bothersome floaters was 18 (lower quartile and upper quartile: 6–60) months. Posterior vitreous detachment (PVD) was found in 20 (42.6%) eyes by ultrasound B scan preoperatively. 27 (57.4%, 27/47) eyes had axial length longer than 26 mm. Before this vitrectomy surgery, phacoemulsification and intraocular lens (IOL) implantation had been performed in 3 eyes for cataract, scleral buckling in 1 eye for RD, retinal photocoagulation in 4 eyes for retinal lattice degeneration or breaks, YAG laser vitreolysis in 10 eyes for vitreous floaters, and vitrectomy in 1 eye for vitreous floaters.

Minimally invasive sutureless 3-port 27-gauge PPV (Constellation; Alcon Laboratories Inc., Fort Worth, TX) was performed under retrobulbar anesthesia (a 50% mixture of 2% lidocaine and 0.75% bupivacaine) by a single surgeon (WRH). The central and peripheral vitreous were removed successively. The PVD induction procedure was chosen by the surgeon's experience. Scleral indentation was performed after adequate vitrectomy to check the peripheral retinal break or retinal degeneration, and photocoagulation would be performed if so. None of the eyes was sutured at the end of the surgery. Vitrectomy was performed for 45 (95.7%) eyes (including 3 pseudophakic eyes), while combined phacoemulsification and intraocular lens (IOL) implantation was performed for the other 2 eyes. During operation, PVD was induced in 6 (22.2%, 8/27) eyes without preexisting PVD. The anterior vitreous was kept in all 42 postoperative phakic eyes.

Comprehensive preoperative ophthalmic examinations, including visual acuity, intraocular pressure (IOP), B scan, spectral domain optical coherent tomography (OCT, Heidelberg, TR-KT-2841, Germany), and panoramic ophthalmoscope (Daytona, P200T), were performed for patients preoperatively and at each examination postoperatively. Subjective refraction for best-corrected visual acuity (BCVA) was performed for patients preoperatively and 3 months or later postoperatively. Patients' satisfaction was evaluated using a questionnaire based on the National Eye Institute validated 25-item Visual Function Questionnaire (NEI VFQ-25) before and within 1 week after surgery. Some modifications of the questionnaire were made on specific problems of floaters when translating to Chinese.

Normal distribution data was presented as mean ± standard deviation, while the nonnormal data was presented as median and quartile range.

## 3. Results

**Figure 1:**
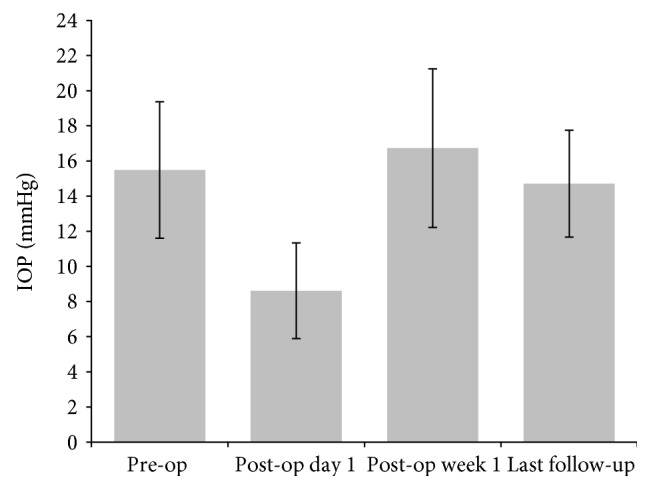
The intraocular pressure (IOP, mean ± standard deviation) at preoperative (pre-op), and different times postoperative (post-op). The last follow-up was 14.0 ± 4.0 months postoperative.

For these 47 eyes, no lens injury, vitreous/retinal hemorrhage, and iatrogenic retinal breaks occurred during operation. The mean follow-up was 14.0 ± 4.0 (range 6.2–24.4) months. The change of IOP at different time points was presented in Figure 1. The IOP at day 1 postoperative was (8.6 ± 2.7 mmHg) apparently lower than other time points (repeated ANOVA, *p* < 0.001). Besides, 28 (28/47, 59.6%) eyes had transient hypotony (IOP < 8 mmHg). All of them recovered at 1 week postoperatively and remained stable at the last follow-up (14.7 ± 3.0 mmHg).

The uncorrected visual acuity (LogMar, median and quartile range) at day 1 and week 1 postoperative was similar than that preoperatively [day 1: 0.79 (0.40, 1.22), week 1: 0.80 (0.35, 1.22), and preoperative: 0.82 (0.30, 1.22)]. Until the last follow-up, the median BCVA was the same as that preoperatively [both were 0.00 (0.00, 0.10)]. Besides, the BCVA of 41 eyes (41/47, 87.2%) remained unchanged or improved. The median BCVA was the same for the 45 eyes without phacoemulsification at the last follow-up and preoperatively [both were 0.00 (0.00, 0.10)].

No intraoperative complications, such as lens injury, iatrogenic retinal break or detachment, and intraocular hemorrhage, were noted. However, postoperative complications occurred in two eyes. One eye (2.1%) had developed *Staphylococcus epidermidis* endophthalmitis at postoperative day 2. After series of treatment, including emergent vitreous tap and silicone oil injection, antibiotics treatment, and silicone oil removal, the patient regained a LogMar BCVA of 0.22 (0.00 preoperatively) at month 5 post operation [16]. One eye (2.1%) with pathological myopia (axial length was 27.08 mm) had developed local RD at month 3 post operation due to a new retinal break. This eye was treated by photocoagulation and laser vitreolysis because of peripheral retinal break 14 months and 7 months before this PPV surgery, respectively. It had preexisting PVD, and no photocoagulation was performed during this vitrectomy surgery since no retinal break was observed. The local RD was treated by further photocoagulation, and the retina was reattached at the last follow-up approximately 6 months later. The BCVA slightly decreased from LogMar 0.00 to 0.05 at the last follow-up.

There were 42 phakic eyes after the vitrectomy surgery. Among these, 10 eyes were diagnosed as age-related cataract (*n* = 3), complicated cataract (*n* = 5), or congenital cataract (*n* = 2) preoperatively. Until the last follow-up (14.0 ± 4.0, range 6.2–24.4 months), none of these phakic eyes developed clinical significant cataract or required cataract surgery for visual acuity improvement. The BCVA of these 42 eyes was 0.10 (−0.08, 0.40) and 0.05 (−0.08, 0.40) preoperative and postoperative, respectively (Wilcoxon test, *p* = 0.15).

**Figure 2:**
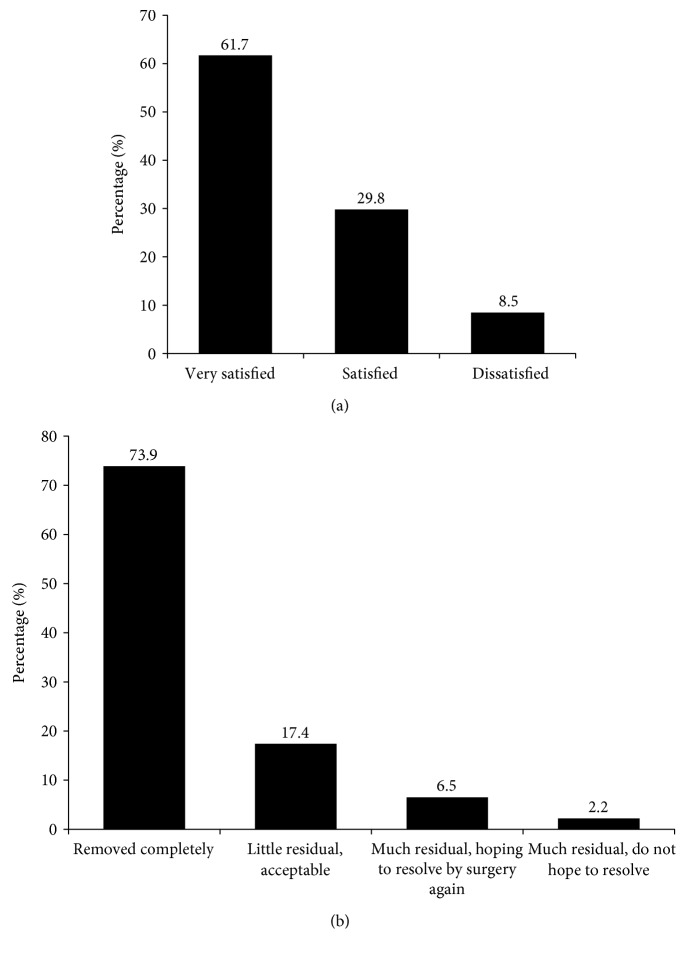
(a) Patients' satisfaction on the vitrectomy surgery (*n* = 47). (b) Patients' subjective feeling on the floaters 1 week after the vitrectomy surgery (*n* = 46, one patient who developed endophthalmitis was unavailable for this question).

There were 91.5% (*n* = 43) of the patients who were very satisfied or satisfied with the surgery outcome, while 8.5% (*n* = 4) were dissatisfied (Figure 2(a)). These patients who were dissatisfied with the surgery were because of postoperative endophthalmitis or residual floaters (*n* = 3). 91.3% (*n* = 42) of the patients felt the floaters were removed completely or only had an acceptable residual, while 8.7% (*n* = 4) did not (Figure 2(b)). One case which developed postoperative endophthalmitis was excluded for this question.

## 4. Discussion

A utility analysis reported that patients with symptomatic floaters were willing to risk a 7% chance of blindness and 11% risk of death to rid themselves off the symptoms [3]. Webb et al. report that among young participants (with an average age of 30 years), 76% reported symptomatic floaters and 33% reported noticeable impairment in vision from floaters [5]. These indicated that a large proportion of patients, especially young patients, are motivated to remove their floaters. Consistently with that report, the patients in this study tended to be young, with a mean age of approximately 35 years, and three quarters were younger than 40 years.

The small gauge vitrectomy surgery was reported to be safe and effective for symptomatic vitreous floaters. de Nie et al. reported that 96.4% of the 110 eyes had no residual on ophthalmoscopy after 20 gauge or 23 gauge (48.2%) vitrectomy for vitreous floaters, with the best-corrected visual acuity significantly improved [8]. However, 10% of the eyes had intraoperative retinal breaks and vitreous hemorrhage, 5.5% had developed cystoid macular edema, 10.9% (three quarters of these were treated by 20 gauge) had developed RD at a mean time of 19.5 months postoperatively, and 50% had developed clinically significant cataract requiring surgery at a mean time of 16.2 months [8]. Mason et al. reported that 92% of the patients found no or only extremely mild symptoms after 25-gauge vitrectomy for symptomatic floaters and the LogMar visual acuity improved from 0.25 to 0.16 [9]. The intraoperative complications include retinal break (7.1%, two thirds of these was preoperative PVD eyes) and vitreous hemorrhage (1.2%). After a mean follow-up of 18 months, 22.5% of the eyes developed a visually significant cataract requiring surgery treatment, while no RD or endophthalmitis was found [9]. Tan et al. found that the intraoperative PVD induction significantly increased the incidence of iatrogenic retinal break (30.5% with PVD induction versus 11.6% without PVD induction, *p* = 0.019) [17]. Considering that the intraoperative PVD induction would possibly increase the incidence of iatrogenic retinal break [17], as well as the postvitrectomy cataract [18], Sebag et al. avoided this procedure during the 25-gauge vitrectomy for symptomatic floaters and found no retinal breaks/detachment, vitreous hemorrhage, or endophthalmitis, and the clinically significant cataracts requiring surgery reduced to 23.5% [10]. Recently, Khan et al. reported that no intraoperative complication for vitreous opacity (*n* = 8) using 27-gauge vitrectomy was found [14]. Based on these reports, the common intraoperative complications, such as iatrogenic retinal break and vitreous hemorrhage, seemed to be reduced by the use of small gauge vitrectomy and without PVD induction procedure.

In this study, preexisting PVD was observed in about 42% of the eyes, which was lower than previous reports (52–74%) [8–10, 17]. This may be due to relatively younger patients in this study. Importantly, the 27-gauge vitrectomy was performed without intraoperative complications in all the symptomatic floater eyes. At the post operation day 1, the IOP was apparently lower than that preoperatively and more than half of the eyes were hypotonic (IOP < 8 mmHg) at day 1 post operation. Postoperative hypotony was reported in 0–25% of sutureless vitrectomy cases [13, 14] and 0–22.6% for vitreous floaters after sutureless vitrectomy with slightly different definition [8, 14, 17]. Mitsui et al. reported that the IOP at day 1 post operation was apparently lower than that preoperatively, with 9.4% of hypotony (<7 mmHg), after 27-gauge vitrectomy for epiretinal membrane with fluid-filled postoperative [13]. The hypotony in this study was comparable with previous reports (14.9% and 4.3% if the hypotony is defined as IOP < 7 mmHg and <6 mmHg, resp.) [8, 14, 17]. Hypotony is usually transient and, in most cases, resolves with conservative measures [13, 14]. In this study, all patients' IOP recovered at week 1 post operation without special treatment, such as systemic steroid use.

One pathological myopia eye (2.1%) had developed RD at month 3 post operation. The rate of RD after 27-gauge vitrectomy was 3.2% [14]. This rate was 0–10.9% for floaters after vitrectomy [8–10, 17, 19], which mostly occurred 12 months after vitrectomy surgery [8, 17, 19]. This rate was lower (0–5.7%) after small gauge vitrectomy (excluded 20 gauge vitrectomy) [8–10]. Though the retinal detachment rate was comparable with previous studies, limited to relatively short follow-up time, the long-term rate was unknown.

In this study, there was no clinical significant postvitrectomy cataract observed in the 42 postoperative phakic eyes. The rate of clinical significant postvitrectomy cataract was 22.5–60.5% after vitrectomy for vitreous floaters [8–10, 17, 19]. The absence of postoperative cataract in this study may be because younger patients were enrolled (i.e., more transparent and younger lens), the anterior vitreous was retained, and the follow-up time was limited.

One eye (2.1%) had developed endophthalmitis at postoperative day 2. This severe postoperative infection was caused by *Staphylococcus epidermidis* (gram-positive cocci), which was a very common type of bacteria existing in healthy human's conjunctival sac. Endophthalmitis after vitrectomy for vitreous floaters is reported sporadically. In two prospective, nationwide studies investigating the endophthalmitis developed after PPV in the UK, Park et al. reported two cases of endophthalmitis following 23-gauge PPV for vitreous opacities, both of which were caused by gram-positive cocci (one was *Staphylococcus epidermidis*) [20, 21]. Henry et al. reported a case of *Staphylococcus caprae* endophthalmitis in a young healthy female after 20-gauge three-port vitrectomy for floaters [22]. The endophthalmitis in this study regained a LogMar BCVA of 0.22 (0.00 preoperatively) at month 5 post operation, after series of treatment.

Limitations of this study included a relatively small sample of patients and relatively short follow-up time. Hence, long-term complications (such as cataract, proliferative vitreoretinopathy, or retinal detachment) and patients' satisfaction were unknown. Furthermore, the contrast sensitivity which may be affected by floaters and vitreous changes [10] was not measured in this study. Further studies with larger sample and longer follow-up time are warranted.

In summary, the 27-gauge vitrectomy was effective and safe for symptomatic vitreous floaters. However, it should be noted that since more healthy young people with excellent vision are trying, even eager to resolve their floaters through seemingly straightforward PPV, potential complications such as cataract, retinal detachment, and even devastating endophthalmitis should be explicitly addressed before this treatment.

## Figures and Tables

**Table 1:** Baseline demographics and characteristics of patients.

Parameters	
Age (years)	34.7 ± 13.5
Male/female	39/8
Right/left	27/20
Symptom time (month^∗^)	18 (6–60)
Pre-op BCVA (LogMar^∗^)	0.00 (0.00–0.22)
Pre-op spherical equivalent (diopter)	−4.95 ± 5.40
Pre-op IOP (mmHg)	15.5 ± 3.9
Axial length (mm)	26.44 ± 2.65
Phakic/pseudophakic	44/3
PVD, *n* (%)	20 (42.6)
Vitreolysis, *n* (%)	10 (21.3)
Vitrectomy, *n* (%)	1 (2.1)
Scleral buckling, *n* (%)	1 (2.1)
Retina Photocoagulation, *n* (%)	4 (8.5)

BCVA: best-corrected visual acuity; IOP: intraocular pressure; pre-op: preoperative; PVD: posterior vitreous detachment. ^∗^The nonnormal data was presented as median and quartile range.
